# Insights into Bacterial Extracellular Vesicle Biogenesis, Functions, and Implications in Plant–Microbe Interactions

**DOI:** 10.3390/microorganisms12030532

**Published:** 2024-03-06

**Authors:** Sarita Pandey, Anaïs Blache, Wafa Achouak

**Affiliations:** 1 CEA, CNRS, Aix Marseille University Lab of Microbial Ecology of the Rhizosphere (LEMiRE), UMR7265 BIAM, F-13115 Saint-Paul-lez-Durance, France; saripandey@gmail.com (S.P.); anais.blache@cea.fr (A.B.); 2 Cyanobacterial Stress Biology and Biotechnology Section, Molecular Biology Division, Bhabha Atomic Research Centre, Trombay, Mumbai 400085, India

**Keywords:** extracellular vesicles, plant–microbe interactions, plant defense mechanisms, interkingdom communication

## Abstract

Plant–microbe interactions play a crucial role in shaping plant health and survival. In recent years, the role of extracellular vesicles (EVs) in mediating intercellular communication between plants and microbes has emerged as an intriguing area of research. EVs serve as important carriers of bioactive molecules and genetic information, facilitating communication between cells and even between different organisms. Pathogenic bacteria leverage extracellular vesicles (EVs) to amplify their virulence, exploiting their cargo rich in toxins and virulence factors. Conversely, beneficial microbes initiate EV secretion to stimulate plant immune responses and nurture symbiotic relationships. The transfer of EV-packed small RNAs (sRNAs) has been demonstrated to facilitate the modulation of immune responses. Furthermore, harnessing the potential of EVs holds promise for the development of innovative diagnostic tools and sustainable crop protection strategies. This review highlights the biogenesis and functions of EVs in bacteria and their importance in plant defense, and paves the way for future research in this exciting field.

## 1. Introduction

In the natural environment, plants coexist with a diverse array of microorganisms, including bacteria, oomycetes, fungi, archaea, protists, and viruses, collectively forming the plant holobiont [1]. These organisms interact with the abiotic environment and each other, influencing the composition of the plant microbiota and its impact on the host [2,3]. Such interactions can range from mutualistic to commensal or even pathogenic, ultimately shaping the plant’s ability to cope with biotic and abiotic stresses [4]. While plants possess their own adaptive mechanisms, they also rely on their microbial partners to survive environmental challenges and defend against pathogens [5]. Root exudates play an important role in the interaction with soil microorganisms [6]. Notably, the communication between plants and microorganisms involves a complex web of interactions, with root exudates playing a crucial role in shaping the microbial community [7,8]. Alahmad et al. [8] demonstrated a connection between root exudates, microbial community composition, assembly processes, and co-occurrence networks in the rhizosphere of pearl millet (PM) lines. They utilized an in situ untargeted metabonomic approach, offering comprehensive insights into the metabolic profiles associated with various compartments of pearl millet and their implications for plant–microbe interactions. Root exudates trigger a beneficial chemotactic response in particular microbial populations attracted by the plant, fostering mutualistic interactions.

Cell-to-cell communication is crucial for interactions among living organisms and the transmission of molecular signals. Extracellular vesicles (EVs) have become crucial facilitators of this communication, allowing for the transportation of cargo over extended distances and ensuring its delivery in concentrated form [9]. The presence of these evolutionarily conserved structures in both prokaryotes and eukaryotes underscores their significance across diverse biological systems. EVs are a heterogeneous population composed of nano to microscale size (20–1000 nm) and derive from various sources [10]. EVs serve as vehicles for transporting proteins, lipids, secondary metabolites, and nucleic acids, including small RNAs (sRNAs). The involvement of sRNAs transported by EVs appears to play a crucial role in host defense, aiding in the prevention of pathogen virulence, while conversely, pathogens utilize them to counteract host defenses. Numerous recent studies have explored the role of sRNAs transported by EVs in plant–fungus interactions, as documented in reviews; however, their involvement in plant–bacteria interactions is still emerging [11]. The communication mediated by EVs can occur through either the uptake of EVs by recipient cells via membrane fusion or endocytosis [12,13], or through the interaction of EV surface receptors with receptors on recipient cells [14].

While EVs have been extensively studied in human cells and human-pathogenic bacteria, and there has been growing research on the role of plant and fungal EVs in host–pathogen interactions, their involvement in plant–bacteria interactions remains largely unexplored. Understanding the significance of bacterial EVs represents a rapidly advancing field. This review offers a comprehensive overview of the biogenesis and functions of extracellular vesicles (EVs), with a particular focus on recent compelling discoveries that underscore their significance as a novel and impactful player in plant–microbe interactions. This highlights an exciting frontier ripe for further exploration and revelation.

## 2. EVs in Plant–Microbe Interactions: Biogenesis and Functional Insights

### 2.1. Bacterial EVs

Bacterial EVs are produced by both Gram-negative and Gram-positive bacteria [15]. Their diameter ranges from 20 to 250 nm. A recent review has shed light on the dual origins of vesicle formation in both Gram-positive and Gram-negative bacteria, as this process can arise from the budding of outer membrane blebs or result from explosive cell lysis triggered by endolysins [16]. In Gram-negative bacteria, extracellular vesicles are classified into two main types: outer membrane vesicles (OMVs), which encapsulate periplasmic components, and outer-inner membrane vesicles (OIMVs), alongside inner membrane vesicles (IMVs) generated during explosive cell lysis. These IMVs harbor cytoplasmic contents, including DNA. To maintain simplicity and clarity, we will collectively refer to both OMVs—IMVs and OIMVs—as “EVs”. Bacterial EVs contain various cargoes such as cell wall components, outer membrane proteins, lipopolysaccharides, phospholipids, proteins, nucleic acids, and secondary metabolites [17]. The contents of EVs may be delivered into animal, plant, and bacterial cells by membrane fusion and/or internalization, and its delivery can be targeted by molecules attached to the outside of vesicles. The production of EVs depends on many factors such as growth stage and stress, peaking at the end of the log phase and the beginning of the stationary phase, and increasing in response to stress [18,19].

EVs of Gram-negative bacteria pathogenic to humans are well characterized, but little is known about microbes interacting with plants. Since the 1980s, EVs have been observed by electron microscopy in cultures of the pathogenic bacteria *Erwinia amylovora* and *Erwinia carotovora* [20]. Several studies have reported the production of EVs by plant pathogens in culture and during plant infection [21,22,23,24]. During infection, EVs have been detected in Gram-negative bacteria interacting with plants and within the plant itself [21], suggesting their involvement in cross-kingdom communication between bacteria and plant cells.

### 2.2. Biogenesis, Secretion, and Uptake of EVs

The biogenesis of EVs in bacteria is still not fully understood, and different models have been proposed to explain their formation [25]. Gram-negative bacteria and Gram-positive bacteria have distinct mechanisms for vesiculation [26,27]. Some proposed models for EV biogenesis include the following [28]:

1. Cell wall turnover: During routine cell wall recycling, lipoproteins between the outer membrane and the peptidoglycans dissociate, leading to membrane protrusion and the release of vesicles into the extracellular space [29].
2. Stress-induced dysfunction: Physical or chemical stress can cause membrane dysfunction, resulting in the accumulation of peptidoglycan fragments or misfolded proteins in the periplasm, triggering vesicle formation [18,19].
3. Cation-induced changes: Cations crossing the electronegative lipopolysaccharide (LPS) layer induce structural changes, leading to differential curvature, fluidity, and charge in the outer membrane. Repulsion between lipopolysaccharides can cause local membrane deformation and shedding [30,31].
4. Conformational changes in outer membrane proteins (OMPs): Changes in the conformation of OMPs can promote vesicle formation. Specific proteins and lipids are locally enriched in areas with high vesicle abundance, while other proteins inhibiting vesiculation, such as lipoproteins, are reduced [30].
5. Explosive cell lysis: A newly proposed mechanism suggests that vesiculation is a result of explosive cell lysis or bubbling cell death, [32], which involves the release of DNA-containing lytic EVs.

A recent review by Juodeikis and Simon in 2022 [33] proposes other EV biogenesis pathways like the weakening of the outer membrane–peptidoglycan linkage or flagellar rotation. It highlights the difficulty in distinguishing between the functions of lytic and nonlytic EVs. 

The rate of vesicle production and the protein content of EVs can vary under different environmental conditions, indicating regulated biogenesis and cargo-sorting processes [18,34]. The determinants, machinery, and rules governing EV formation and the incorporation/exclusion of specific proteins into EVs are still areas of active investigation [35]. 

### 2.3. Functions of Bacterial EVs

A variety of EV functions have been reported, which are related to interbacterial as well as host–bacteria interactions. A recent review by Toyofuku et al. [16] describes the functions of bacterial EVs well. In various aspects, the formation of bacterial EVs is in favor of the bacteria and their host partner. They are associated with several crucial functions, such as (i) cell–cell communication, (ii) the formation of biofilms and horizontal gene transfer to survive environmental stressors [36], (iii) transport and delivery, and (iv) stress response [17,18].

**Figure 1:**
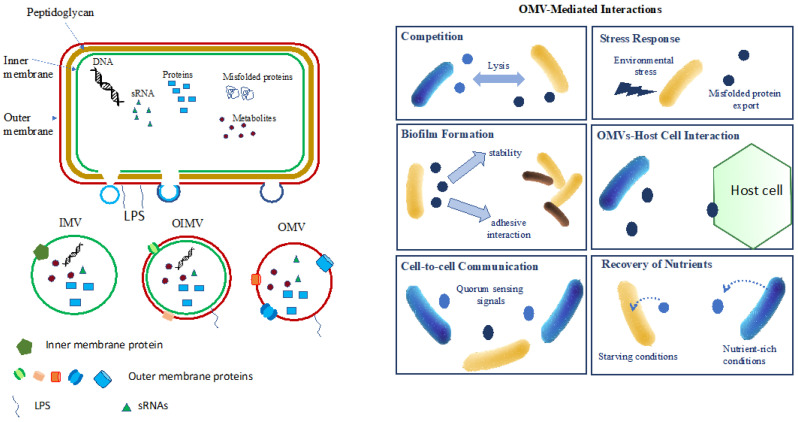
Bacterial vesicle (EV) production and mediated functions. (**left**) Production of EVs: EVs are produced through outer membrane blebbing, wherein portions of the outer membrane (OMV), outer–inner membrane (OIMV) bud off, or inner membrane (IMV) result in the formation of vesicles containing a cargo of proteins, RNA, and metabolites, or even DNA for OIMVs and IMVs, as reported by Toyofuku et al. [16]. (**right**) EV-mediated functions: EVs play diverse roles facilitated by their unique cargo, including competition, stress response, biofilm formation, host interaction, cell–cell communication, and nutrient recovery.

Figure 1 presents a comprehensive overview of the various types of vesicles generated by Gram-negative bacteria, along with their cargo and primary functions.

### 2.4. Cell-Cell Communications and Quorum Sensing (QS)

EVs play a crucial role in cell-to-cell communication, particularly in the distribution of quorum sensing (QS) signals among bacterial populations [37]. QS signals play a pivotal role in the regulation of virulence factors in numerous pathogenic bacteria. They also have a significant impact on the modulation of beneficial traits in the microbiota associated with plants. QS molecules are often hydrophobic, like *Pseudomonas* Quinolone Signal (PQS) of *Pseudomonas aeruginosa* [37], C16-HSL of *Paracoccus denitrificans*, and CAI-1 of *Vibrio harveyi*. By packaging hydrophobic QS molecules into vesicles, EVs solubilize and stabilize these signals, allowing their passage through the lipopolysaccharide layer of producing and receiving cells [38,39]. QS signals can also influence EV production in other bacteria. PQSs produced by *P. aeruginosa* can induce EV formation in other species such as *Escherichia coli*, *Burkholderia cepacia*, and *Bacillus subtilis* [36,40]. In a recent study, Fan et al. [41] illustrated that the α-hydroxy-ketone compound Legionella autoinducer-1 (LAI-1) is secreted via EVs. This secretion mechanism facilitates interbacterial communication and interactions with eukaryotic host cells.

Furthermore, EVs may facilitate the horizontal transfer of antibiotic resistance genes between bacteria [42,43], enabling the spread of resistance proteins to neighboring cells [44]. This transfer can occur within or between species through EV fusion with recipient cell membranes [45].

### 2.5. Biofilm Formation

Bacterial EVs play a significant role in biofilm formation, an essential survival strategy for bacteria. EVs contribute to biofilm formation and maintenance by mediating adhesive interactions, facilitating nutrient delivery to cells within the biofilm matrix, and enabling the long-range transport of molecules involved in virulence and antibiotic resistance [46,47,48].

Furthermore, EVs can act as offensive tools by lysing competing bacteria, thereby enhancing the competitive advantage of the producing bacteria [49]. In the context of the plant microbiota, EVs may influence the ability of competing microbes to adapt to the host environment by promoting cell lysis [28]. This inter-microbial competition within the root microbiota can involve EVs, which exhibit direct antifungal activities and contribute to the establishment of bacterial commensals in roots, thus protecting plants from harmful filamentous eukaryotes [50,51].

Additionally, EVs derived from *Xylella fastidiosa* ssp. *pauca* contain XfYgiT, a component of the toxin–antitoxin system known to regulate biofilm formation and contribute to the survival of *X. fastidiosa* ssp. *Fastidiosa* in planta [24].

These findings highlight the significant role of bacterial EVs in biofilm formation and their potential as mediators of inter-bacterial interactions and plant–microbe relationships within complex microbial communities.

### 2.6. Transport and Delivery

For pathogenic bacteria, toxins and virulence factors are transferred to eukaryotic target cells by EVs [52]. Extracellular vesicles produced by *Campylobacter jejuni*, carrying cytolethal distending toxin, interact with host cell glycans, triggering cell cycle arrest in the host cells [53].

Proteomic analysis of EVs produced by various strains of *Xanthomoas campestris* and *Pseudomonas syringae* revealed the presence of virulence determinants as cellulase and xylosidase), type II or III components (AvrA1 and HopI1) produced by *P. syringae*, which are known to suppress plant immune responses and secreted proteins [21,54]. However, it remains unclear whether microbial EVs release their cargo into the extracellular space or have the ability to interact with plant cells to deliver their cargo into the cytosol. 

Biller et al. [55] analyzed the cargo of *Prochlorococcus*, a marine cyanobacterium, using omics techniques. EVs produced by this marine bacterium contain lipids (polar lipid IPLs), pigments (carotenoids and plastoquinone), proteins implicated in transport (porins and transporters), peptidase, hydrolase, and chaperones, suggesting various functions for these vesicles, such as the dissipation of oxidative stress and the transport of damaged or hydrophobic molecules. The presence of sugars like triose and tetrose suggests that the vesicles may be a carbon source for marine organisms [55]. These functions may enhance the bacterial fitness in the rhizosphere, a dynamic environment where plants and other microorganisms can produce antimicrobial compounds, and where potential exposure to toxic substances, such as pesticides, is not uncommon. Moreover, the nutritional content within the EVs could be a valuable resource for bacterial survival in the oligotrophic soil environment, especially in the absence of the host plant.

Moreover, EV-associated DNA has been implicated in facilitating horizontal gene transfer (HGT) among bacteria, which encompasses the dissemination of antibiotic resistance genes within and across bacterial species. A study by Johnston et al. [56] indicates that *P. aeruginosa* EVs have the capability to promote the spread of antibiotic resistance genes. This mechanism facilitates the survival of susceptible bacteria during antibiotic treatment.

Research conducted by Li et al. [57] has revealed that avian pathogenic *E. coli* EVs facilitate the horizontal transfer of blaCTX-M-55. This discovery sheds light on how resistance is potentially proliferated within the poultry industry, underscoring the necessity for strict limitations on antibiotic use in poultry farming. The phenomenon of EV-mediated gene transfer has also been observed in Klebsiella pneumoniae, where plasmids carrying resistance genes are transferred via EVs, as demonstrated by Dell’Annunziata et al. [58].

### 2.7. Stress Response

Bacterial EVs are a crucial strategy employed by bacteria to survive environmental stresses. They sequester and export misfolded toxic proteins generated during heat stress [18], providing protection against complement components [59], long-chain alcohols, metal chelators [46], and antimicrobial peptides [47,60]. 

Membrane vesicle production is highly influenced by various stressors, such as oxidative stress, UV radiation, nutrient deprivation, pH, temperature, hydration, and antibiotics [61]. These stressors can directly induce the export of misfolded proteins or induce outer membrane blebbing following changes in outer membrane composition (Figure 1). 

Lima et al. [62] present compelling evidence suggesting that bacteria from diverse ecological niches release abundant EVs in response to copper exposure. Their findings, particularly with the cyanobacterium *Synechocystis*, indicate that EV release in bacteria offers a novel mechanism for copper secretion, providing insights into alternative pathways for bacterial metal resistance. 

EVs contribute to the management of iron scarcity, a crucial factor, particularly in the case of pathogens such as *Mycobacterium tuberculosis*. These EVs carry mycobactin, a hydrophobic siderophore, which helps sequester iron from the environment and enhances pathogen survival during infection [63]. In the extracellular milieu of *P. aeruginosa*, the formation of the PQS-Fe3+ complex occurs, which is efficiently transported by EVs. These EVs serve as carriers, facilitated by the T6SS effector protein TseF, allowing the PQS-Fe3+ complex to hitchhike onto them. Subsequently, the complex gains access to the bacterial periplasm through the outer membrane receptors OprF and FptA, as detailed by Zhang et al. [64].

Furthermore, EVs produced by *Corynebacterium glutamicum* also function as extracellular iron carriers, enabling iron uptake through a mechanism that operates independently of membrane-associated proteins or siderophores, as demonstrated by Kawashima et al. [65]. These intricate processes hold significant implications in environments such as the rhizosphere, where iron availability is often constrained. The investigation into the acquisition of extracellular vesicle-associated iron by EV-producing bacteria, other microbial entities, or the plant host represents a promising avenue for further exploration.

## 3. Role of EVs and sRNA in Plant–Microbe Interaction

### 3.1. Plant–Microbe Symbiosis

Research has documented the presence of EVs in rhizobia, with their isolation achieved under living conditions [66]. Moreover, characterization efforts have utilized proteomics to compare the expression profiles of periplasmic space proteins with those found in the EVs of *Rhizobium etli* [67], along with investigating the protein profiles of EVs from *R. etli* both before and after naringenin induction [68]. The role of EVs in symbiosis establishment, such as EVs produced by *Sinorhizobium fredii* HH103, plays a role in the establishment of rhizobium–soybean symbiosis. The treatment of soybeans with EVs induced the expression of nodulation genes, suppressed plant defense genes, and affected root development [69].

**Figure 2:**
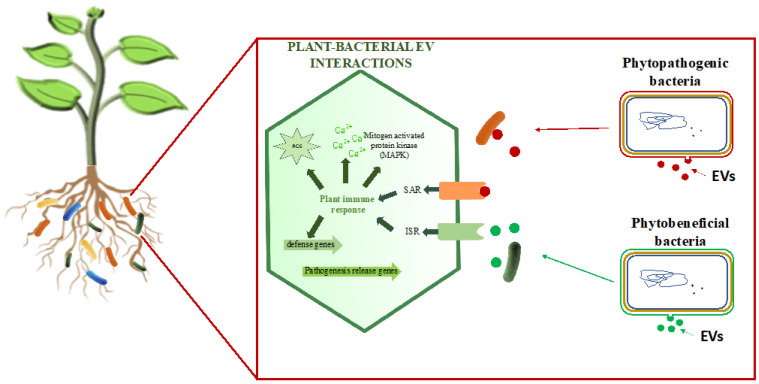
Extracellular vesicle-mediated interkingdom communication in the rhizosphere. This figure illustrates the activation of systemic acquired resistance in plants by pathogenic bacteria, as well as the induction of induced systemic resistance (ISR) in plants by beneficial bacterial EVs.

Overall, EVs contribute to interorganismic signal and nutrient exchange, enhancing symbiotic interactions between plants and microbes. Figure 2 depicts the role of both phytobeneficial and phytopathogenic bacterial extracellular vesicles (EVs) in facilitating interkingdom communication within the rhizosphere.

### 3.2. EV-Packed sRNA and Pathogenicity

EVs produced by pathogenic bacteria are enriched in toxins and virulence factors, which are internalized into host cells to promote pathogenicity [9,18,19,30,70]. Proteomic studies have revealed that EVs from plant pathogenic bacteria contain plant cell wall-degrading enzymes, protein secretion machinery components, effectors, nucleic acids inducing plant immune responses, and various virulence factors [21,54,70,71]. Some of these nucleic acids are small RNAs (sRNAs). Employing a blend of infrared and circular dichroism spectroscopies, Turnbant et al. [72] showcased, in *E. coli,* the translocation of Hfq from the inner membrane into the periplasm, followed by its exportation within EVs, potentially binding to sRNAs. sRNAs hold the capability to directly enter host cells, thereby silencing host defense genes and attenuating host immunity. Numerous studies have affirmed that the host immune responses, provoked by sRNAs encapsulated within EVs, facilitate bacterial growth and host infection in vivo [73,74,75,76]. Recently, Wu et al. reported on the role of sRNA Xosr001 in regulating immunity in rice plants. Xosr001, found abundantly in extracellular vesicles (EVs) produced by *Xanthomonas oryzae* pv. *oryzicola* (*Xoo*), is delivered to rice leaves via EVs, where it suppresses OsJMT1 expression [77]. This suppression leads to a decrease in MeJA accumulation and compromised stomatal immunity. The application of Xosr001 to OsJMT1-HA-OE transgenic rice results in effective suppression of OsJMT1 expression, ultimately reducing stomatal immunity and enhancing susceptibility to disease, underscoring Xosr001’s role in undermining rice’s defense mechanisms [77]. Furthermore, recent studies have shown that sRNA4518698, sRNA2316613, and sRNA809738 emerged as the top three abundant sRNAs within EVs, originating from non-coding RNAs of *P. aeruginosa* [78]. Notably, these sRNAs were securely encapsulated within the interior of EVs, exhibiting exceptional resilience against external RNase cleavage. Intriguingly, transfecting synthetic sRNA4518698, sRNA2316613, or sRNA809738 led to a reduction in the expression of innate immune response genes in RAW264.7 cells, unveiling a mechanism by which EVs modulate host responses through the transfer of bacterial sRNAs [78]. 

EVs serve as crucial mediators in the interaction between bacterial sRNAs and the host, offering novel insights for managing bacterial infections [79]. Methods and protocols detailing the characterization of EVs and their sRNAs are available in the literature [80,81]. 

### 3.3. Bacterial EVs: Activating Signal for Plant Immune Response 

EVs produced by pathogenic bacteria can act as activating signals for plant immune responses [82,83]. Pathogenic bacteria often induce the expression of the isochorismate synthase 1 (ICS1) gene in the host, which encodes an enzyme that catalyzes the production of salicylic acid (SA), a plant immune signal for systemic acquired resistance [84,85]. EVs from *X. campestris* pv. *Vesicatoria* (*Xcv*), *X. campestris* pv. *Campestris* (*Xcc*), and *Xoo* and virulence factors purified from these EVs have been shown to trigger immune responses in plants, including callose deposition, the alkalinization of the medium, increased transcription of pattern recognition receptors, the activation of Mitogen-Activated Protein Kinase (MAPK), and the release of reactive oxygen species [21,22]. Alterations in crucial receptors like the plant elongation factor receptor (EFR), flagellin receptor (FLS2), or brassinosteroid-insensitive 1–associated kinase (BAK1) co-receptor had minimal effect on the immune priming ability of *Xcc* EVs [86]. These findings suggest that *Xcc* EVs elicit a widespread transcriptional shift in *Arabidopsis*, stimulating the activation of multiple immune pathways. *Xcc* EVs were shown to induce the expression of multiple WRKY transcription factors. This transcriptional modulation holds promise for enhancing the plant’s defense mechanisms against bacterial infections [86]. Tran et al. [87] elucidated that the structured lipid composition of EVs enables direct interaction and integration of *Xcc* EVs into the *Arabidopsis* plasma membrane (PM), thereby enhancing its lipid organization. Moreover, their study showcased that *Xcc* EVs elevate both cellular endocytosis rates and PM lipid organization, effectively fortifying plants against electrolyte leakage and bacterial infections.

Even, non-pathogenic bacteria such as *Pseudomonas fluorescens* can also activate plant immune responses at low levels [88]. The immune activation is triggered by Microbe-Associated Molecular Patterns (MAMPs) and induces a systemic immune response via SA-independent pathways [88]. Notably, pathogens such as *P. syringae* can overcome plant defenses using the type III secretion system (T3SS) [89], while other bacterial secretion pathways also play a role in plant–microbe interactions [90]. EVs of *P. fluorescens* can activate Induced Systemic Resistance (ISR) in *A. thaliana* and reduce the development of *P. syringae* [91]. 

Plants have developed two forms of innate immunity in response to infection: pathogen-associated molecular pattern (PAMP)-triggered immunity (PTI) and effector-triggered immunity (ETI), also known as R-gene-based immunity [92]. PTI is initiated by the recognition of conserved microbe-associated molecular patterns (MAMPs) by receptor-like kinases (RLKs) and receptor-like proteins (RLPs) on the cell surface, leading to immune responses [93]. The response includes the production of reactive oxygen species (ROS), the activation of defense genes, an increase in calcium concentration, callose deposition for cell wall thickening, MAPK activation, and the expression of pathogenesis-related genes [94,95,96]. ETI, on the other hand, relies on the specific recognition of defense molecules produced by plant resistance genes or pathogen effectors. Pathogens deliver effector molecules to inhibit the PTI response, prompting plants to activate the intracellular receptor proteins containing a nucleotide binding domain (NBD) and a leucine-rich repeat (LRR) domain (NLR) for ETI [92]. During microbial infection, the host immune responses require a specific reprogramming of gene expression and communication between hosts and microbes.

Lipopolysaccharide (LPS) and elongation factor Tu (EF-Tu) are prevalent components of bacterial EVs, and they function as MAMPs activating pattern-triggered immunity (PTI) upon recognition by plant-encoded immune receptors [22,54]. EVs carrying these MAMPs, such as EVs from *Xcc*, induce ROS production, ion release, and defense gene expression in *A. thaliana* through the EF-Tu receptor (EFR) [22,86]. EVs from other Gram-negative bacteria, including *Xoo*, *P. syringae* pv. *tomato* DC3000, and *Acidovorax citrulli* M6, also induce defense gene expression [22]. 

The ability of bacterial EVs to induce plant immune responses demonstrates their immunogenic potential, and their interaction with cell-surface-localized receptors EFR, BAK1, and SOBIR1 suggests direct interaction with plant cells [87,97].

### 3.4. Plant–Microbiota Vesicle Interactions

The rhizosphere microbiota may play an important role in plant growth and protection against pathogens mediated by beneficial bacteria inhibiting pathogen growth. Xia et al. [98] demonstrated that the host plant, soybean, and soil microbes complement the thiamine auxotrophy of the oomycete pathogen Phytophthora sojae. Conversely, myxobacteria inhibit *Phytophthora* growth by secreting thiaminase I CcThi1 into the extracellular environment via outer membrane vesicles [98]. 

This emerging area of research holds tremendous potential for understanding their impact on plant growth and health, particularly in the rhizosphere. As previously noted, both prokaryotes and eukaryotes produce EVs. In the rhizosphere, where plants, bacteria, and fungi coexist, their EVs may facilitate the essential molecular dialogue for these interactions [99,100]. The contents of these EVs hold the potential to promote inter-kingdom communication, thus impacting microbiota assembly and competitive processes in parallel. By delving deeper into the composition and impacts of EVs originating from both the host and its associated microbiota, within the context of the rhizosphere, we may unlock invaluable insights into the intricate dynamics governing microbiota assembly, inter-microbial relationships, and the nuanced interplay between plants and microbes).

**Figure 3:**
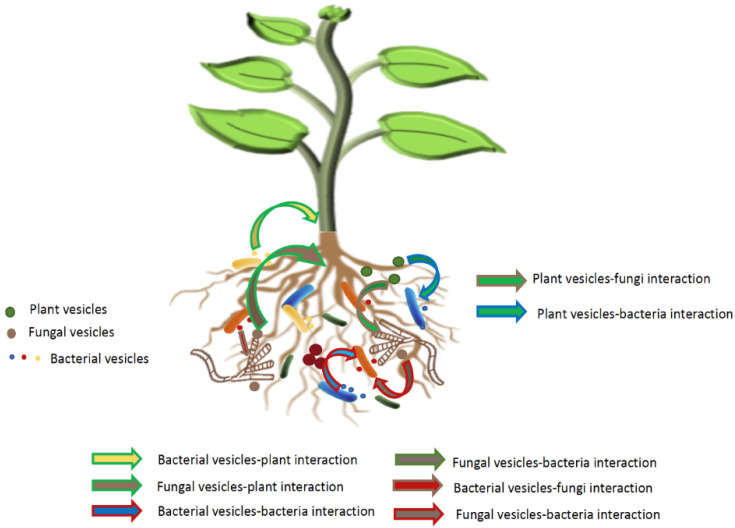
EV-mediated interaction network in the rhizosphere. This figure depicts the intricate interaction network involving EVs in the rhizosphere: plant EVs and recruitment of root-associated microbiota; microbe EVs and microbiota assembly; synergic and antagonistic interactions.

Figure 3 illustrates the diverse interactions potentially facilitated by the extracellular vesicles of the host plant and its microbiota.

## 4. Perspectives

In line with research focusing on the role of EVs in human/animal–bacteria interaction, we believe that by further exploring the cargo of EVs released by various microbial species involved in plant–microbe interactions—whether beneficial microbes or pathogens—we can gain valuable insights into their strategies for manipulating host plants and influencing the plant immune system.

Plant, fungi, and bacteria are known to produce EVs. One intriguing area of research lies in the crosstalk between plant-derived EVs and microbial EVs. Exploring how these EVs communicate and exchange cargo with one another could shed light on the underlying mechanisms of the symbiotic relationships, pathogen recognition, and modulation of plant immune responses.

Nonetheless, the primary challenge lies in extracting vesicles from various sources and accurately distinguishing them based on their origins, despite their nanoscale size. Additionally, discerning their cargoes poses another significant hurdle. Thus, the imperative task of identifying specific biomarkers for extracellular vesicles (EVs) based on their origins becomes apparent.

By manipulating the cargo of EVs or engineering EVs to optimize plant–microbe interactions by selectively loading specific molecules into EVs, we may unlock the potential for novel strategies in plant protection and improved crop production.

It is important to explore the contribution of microbial EVs in microbe–microbe interactions and the intricate dynamics of microbiota–plant interactions under natural conditions.

Continued exploration and a deeper understanding of the intricate interplay between plants and their associated microbiota through EV-mediated communication will contribute significantly to advancements in agriculture, crop protection, and ecological sustainability.
